# Comparative pharmacokinetics of osmotic-controlled and immediate-release Eperisone tablet formulation in healthy human subjects using a sensitive plasma LC-ESI-MS/MS method

**DOI:** 10.1038/s41598-020-58801-1

**Published:** 2020-02-05

**Authors:** Kamran Ahmed, Muhammad Harris Shoaib, Rabia Ismail Yousuf, Fahad Siddiqui, Faaiza Qazi, Javeria Iftikhar, Farrukh Rafiq Ahmed, Muhammad Iqbal Nasiri

**Affiliations:** 1 Department of Pharmaceutics and Bioavailability and Bioequivalence Research Facility, Faculty of Pharmacy and Pharmaceutical Sciences, University of Karachi, Karachi, 75270 Pakistan; 2 Department of Pharmaceutics, Faculty of Pharmacy and Pharmaceutical Sciences, University of Karachi, Karachi, 75270 Pakistan; 3 Department of General Surgery, Hamdard College of Medicine and Dentistry, Hamdard University, Karachi, 75300 Pakistan; 4 Present Address: Hamdard Institute of Pharmaceutical Sciences, Hamdard University, Islamabad Campus, Islamabad, Pakistan

**Keywords:** Drug delivery, Medical research

## Abstract

To evaluate and compare the pharmacokinetic (PK) characteristics of a newly developed oral osmotically controlled drug delivery system of Eperisone 150 mg tablets with Eperisone immediate release (IR) marketed tablet brand as a reference formulation. It was a single dose, two treatment, two sequence, randomized, crossover study, involving 12 healthy human subjects. A modified, sensitive LC-ESI-MS/MS method was developed and validated as per FDA guidelines for estimation of Eperisone in plasma using a simple extraction and quick protein precipitation method. Non-compartmental pharmacokinetic model was used for PK analysis. Results were statistically compared using logarithmically transformed data, where p > 0.05 was considered as non-significant with 90% CI limit of 0.8–1.25. The bio-analytical method used for estimating drug plasma concentration was found to be simple, selective, linear, accurate and precise with 0.01 ng/ml as limit of detection. The comparative PK analysis revealed an insignificant difference in AUC_0-∞,_ AUC_0-t,_ V_z_/F, Cl/F and t_1/2λz_, whereas a significant difference in C_max_, T_max_ and MTTs were found. The relative bioavailability of Eperisone osmotic tablet was 109.7%. The osmotic controlled release drug formulation was found to release Eperisone for an extended period with less inter individual fluctuation in pharmacokinetic variables.

## Introduction

Sustained release formulations are one of the famous type of controlled release drug delivery system. The drug release rate from these products may depend on the pH, food and other physiological factors of gastrointestinal tract (GIT). Thus, the pharmacokinetics (PK) of drugs showing variable inter personal pharmacokinetic characteristics cannot be controlled and predicted by designing sustained release product of the drug. Osmotically controlled drug delivery system, in addition to the benefits of sustained release products (decreased dosing frequency, enhanced patient compliance and least side effects) also delivers the drug at a relatively constant rate (zero-order) without being considerably affected by the pH, food and other hydrodynamic conditions of the GIT. Hence, the drugs with variable PK characteristics can be designed into osmotically controlled drug delivery system to avoid the inconsistency in PK variables^1–4^.

Eperisone belongs to BCS class I, possessing high aqueous solubility and high permeability. Therapeutically, Eperisone is an anti-spastic agent which provide muscle relaxant activity by acting in central nervous system. It is supposed to block both calcium and sodium voltage-gated channels present in spinal cord, reducing the gamma-efferent firing in spinal cord structures and hence decreasing the spinal cord activities. Eperisone also possess some vasodilator activity and antinociceptive effects^5–8^. Although the use of centrally acting muscle relaxants is associated with undesirable side effects like drowsiness, dizziness and ataxia, but still they are widely prescribed alone or in combination with analgesics for the management of muscle spasms and myalgias, more commonly lower back pain^9–12^. Eperisone because of its mechanism of action, is comparatively devoid of the undesirable side which are usually associated with intake of centrally acting muscle relaxants^7,8,13–15^. It is usually administered at an oral dose of 150 mg/day in three divided doses. It has short biological half-life (1.6 to 1.8 hrs) with a considerable variation in inter-personal pharmacokinetics^16–18^. The previous published data regarding the pharmacokinetics of Eperisone indicated rapid absorption from the GIT with time to reach the maximum plasma concentration range of 0.3–2 hours. But the peak plasma concentration and area under the plasma concentration curve reported by different workers were found to vary (C_max_ = 0.80–44.8 ng/ml, AUC_0-∞_ = 1.16–76.1 ng/ml × h) widely. Interpersonal PK variation was also noticed within a study, specifically in case of C_max_^16,18–23^_._

The characteristics of Eperisone like high aqueous solubility, short duration of activity and variability in PK characteristics make it an excellent candidate to be designed as an oral osmotic drug delivery system. Hence, oral osmotically controlled drug delivery system of Eperisone was developed and optimized by Ahmed *et al*.^24^. In the study each osmotic tablet containing 150 mg of Eperisone was supposed to be administered once daily. The optimized osmotically controlled drug delivery system of Eperisone was found to release the drug in zero-order pattern, without being significantly affected by pH and agitation intensity of dissolution medium. The details of development, evaluation and optimization of this system and effect of formulation variables on drug release profiles was reported in 2018^24^.

The bio-analytical method employed for the detection of plasma Eperisone should be sensitive enough to determine the concentration in terminal elimination phase, owing to C_max_ reported as low as 0.80 ng/ml. Several bio-analytical methods for the estimation of Eperisone in plasma have been developed and reported. These methods utilized the technique of LC-MS/MS or LC-ESI-MS to estimate the concentration of Eperisone in biological fluids. These methods utilized the complex and time consuming liquid-liquid extraction techniques for extraction of Eperisone from plasma^16,22,25^.

In the current study a sensitive, simple and less time consuming bio-analytical method for determination of Eperisone in human plasma, utilizing chromatography-electrospray ionization-mass spectrometry (LC-ESI-MS/MS) technique was developed by modifying the previously reported methods. The method was validated and then applied in the comparative PK analysis of the optimized osmotic formulation of Eperisone in local population with immediate release tablets ingested in a single dose of 150 mg.

## Materials

### Chemicals used in bio-analysis

Acetic acid was purchased from Merck, KGaA, Germany. Acetonitrile and Methanol (LC-MS grade) were purchased from VWR International, Fontenay-sous-Bois, France. Tizanidine and Eperisone were gifted by Ali Gohar Pharmaceuticals Private Limited and Platinum Pharmaceuticals, Karachi, Pakistan, respectively.

### Pharmacokinetic study products

Osmotically controlled formulation of Eperisone was selected as test product. The development, evaluation and optimization of Eperisone osmotic formulation has already been published previously^24^. The optimized core tablet was composed of Eperisone (150 mg), microcrystalline cellulose (MCC, filler/binder), sodium chloride (NaCl, osmogent), colloidal silicon dioxide (glidant) and magnesium stearate (lubricant and antiadherent). The core tablets were coated with Opadry® CA (Coloron Limited, Kent, England) with a weight gain of 8% w/w and an orifice having a diameter of 0.8 mm was developed in centre at one side of the coated tablets^24^.

Immediate release tablets sold under the brand name of Smur (50 mg/tablet), manufactured by Barrett Hodgson Pvt. Ltd, Karachi, Pakistan was selected as a reference product.

## Methods

### Bio-analytical method

#### Preparation of standard solutions and quality control (QC) samples

The standard stock solution of Eperisone and Tizanidine (internal standard) having a concentration of 100 ng/ml and 10 µg/ml, respectively were freshly prepared. Solution containing 1% v/v acetic acid in deionized water and 1% v/v acetic acid in methanol in a ratio of 50:50% was used as solvent. The Quality Control samples (QCs) having Eperisone concentration of 0.4 ng/ml, 3 ng/ml and 8 ng/ml were coded as QCL (quality control low), QCM (quality control medium) and QCH (quality control high), respectively. These QCs were prepared with the help of a standard stock solution of Eperisone in drug free human plasma.

#### Chromatographic and mass spectrometric conditions

**Figure 1:**
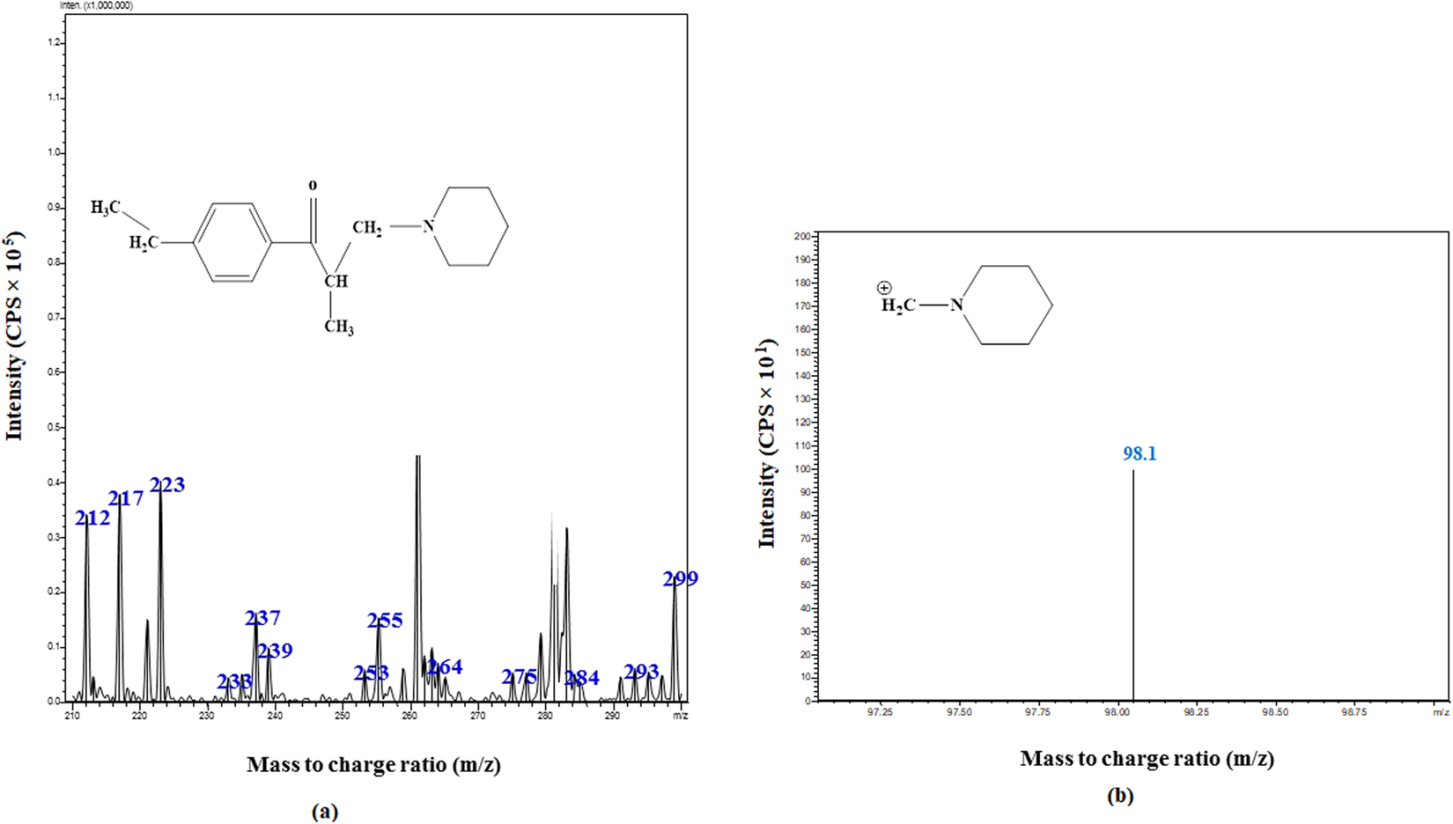
MS/MS spectra showing (**a**) Precursor ion and (**b**) Product ion of Eperisone.

**Figure 2:**
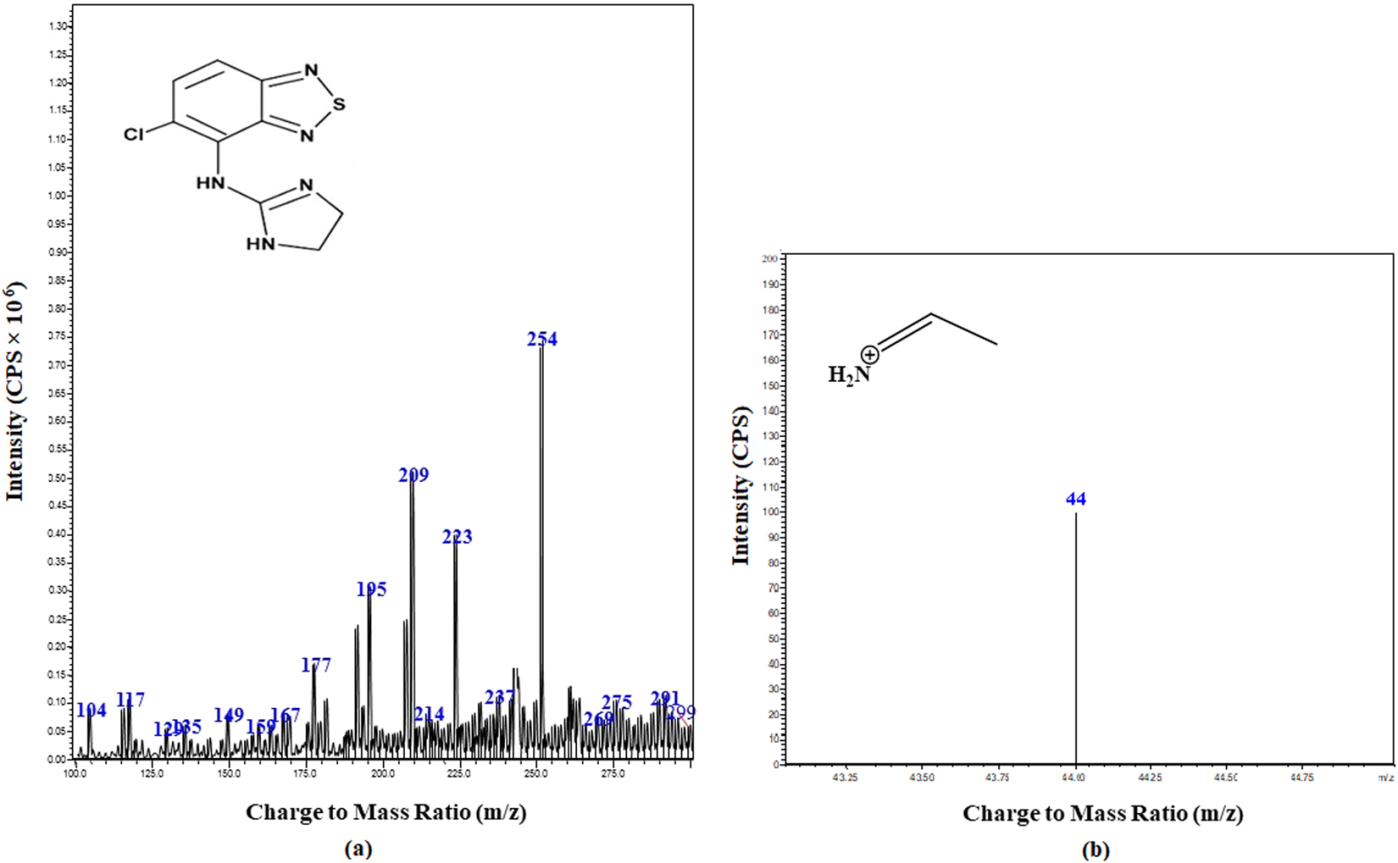
MS/MS spectra showing (**a**) Precursor ion and (**b**) Product ion of Tizanidine.

Eperisone was analyzed by a liquid chromatograph mass spectrophotometer (LCMS-8040, Shimadzu, Kyoto, Japan) using a LC column (Shim-pack XR-ODS-228-41606-92, 3 × 50 mm, 2.2 µm, Shimadzu, Kyoto, Japan), isocratic pump (LC-20 AD, Shimadzu, Kyoto, Japan), autosampler (SIL-20 AC, Shimadzu,Kyoto, Japan), a column heater (CTO-20A), and a degasser (DGU-20A5R). The mobile phase was prepared by modifying the composition of the mobile phase reported by Jeoung *et al*.^16^. Mobile phase having a composition of 1% v/v acetic acid in deionized water and 1% v/v acetic acid in methanol in a ratio of 50:50% was prepared, filtered (Bio-Care Lab Ware, Islamabad, Pakistan) under vacuum through a membrane filter (0.45μm) and degassed by sonication (Ultra-Sonic Bath: Elma, Singen, Germany). The mobile phase was pumped at a flow rate of 0.2 ml/min. The injected volume of samples was 5 μl and the column was maintained at a temperature of 40 °C. Positive ionization mode was selected, and the ions were monitored in the multiple reaction monitoring (MRM) mode with dwell time of 100 milliseconds and collision energy of 17 V and 27 V were set for Eperisone and Tizanidine, respectively. The mass spectrometer was operated at ESI interface and the following operating parameters were set:

Drying gas flow rate was 5 L/min at 250 °C, nebulizing gas flow rate was 3 L/min at 15 psi, desolvation line (DL) temperature and heat block temperature were 250 °C and 400 °C, respectively. The precursor and product ion pairs were monitored at 260.2 → 98.1 m/z and 254 → 44 m/z for Eperisone and Tizanidine (as shown in Figs. 1 and 2), sequentially. The data obtained was processed and analysed by using the Lab solution software (Shimadzu, Koyoto, Japan).

#### Sample preparation and determination of drug in human plasma

In 0.5 ml of volunteer plasma, 50 μl of internal standard (10 μg/ml) and 0.5 ml of acetonitrile (deproteinating agent) were added. The contents were mixed for 2 minutes using a vortex mixer (Stuart, Leicestershire, England.), centrifuged (Mikro 120, Hettich, Germany) at a speed of 10,000 rpm for 10 minutes. The supernatant clear liquid layer was separated, filtered through a 0.22 μm nylon filter (0.22 µm pore size, 13 mm dia, Allpure, Hampshire, England) with the help of a syringe and placed in an auto sampler (SIL-20 AC, Shimadzu,Koyoto, Japan).

#### Method validation

The method selectivity, linearity, accuracy, precision and stability were validated as per FDA guidelines^26^.

Selectivity. The selectivity was determined as per FDA guidelines by comparing the response of extracted blank plasma samples (taken from 6 different sources) with the response of spiked matrix samples containing Eperisone in following concentrations: QCL (0.4 ng/ml), QCM (3 ng/ml) and QCH (8 ng/ml).

Stability. The stability of Eperisone was determined under different analytical and environmental conditions. In the determination of stability, plasma samples containing Eperisone in concentration of QCL (0.4 ng/ml), QCM (3 ng/ml) and QCH (8 ng/ml) were prepared. The results of stability were obtained in terms of % recovery of analyte.

Solutions with a defined concentration of 100 ng/ml Eperisone and 10 µg/ml Tizanidine (IS) were prepared and analysed to obtain peak areas in mobile phase at the same day of preparation of stock solution. After a week fresh dilution was again prepared and analysed, the calculated area was compared with the peak areas of initial samples to obtain % recovery after a week. Same procedure was repeated for further three weeks (Table 1).

**Table 1:** Stock Solution Stability of Eperisone and Tizanidine in plasma.

Stock Solution Stability									
Eperisone					Tizanidine				
Conc.	Recovery (%)				Conc.	Recovery (%)			
100 ng/ml	Week one	Week two	Week three	Week four	10 µg/ml	Week one	Week two	Week three	Week four
1	96.80	98.20	97.83	95.80	1	98.49	97.70	99.00	95.50
2	98.28	99.20	96.38	96.50	2	97.20	98.20	96.00	96.80
3	97.42	97.20	97.80	98.80	3	99.00	95.50	99.68	97.00
Mean	97.5	98.2	97.1	96.15	Mean	98.23	97.13	98.23	96.43

In order to determine auto sampler stability, the QCs (low, medium and high) were extracted from plasma, placed in an auto sampler and injected three times at 0 hours and 6 hours. The % recovery was then calculated.

The long-term stability of Eperisone was determined by preparing the QCs and storing them at −20 °C. Twelve samples of each concentration were prepared. Three samples from each QCs were analysed on the day of collection of 1st plasma sample from volunteer and were coded as the zero-week samples. The rest of the samples were frozen at −20 °C. After 1 week again three samples from each QCs were analysed, while the rest of the samples were frozen again at −20 °C. This procedure was repeated at week 3 and week 4 (the last day of sample analysis). The integrity of drug was obtained by comparing the response of QCs at different time intervals with response of QCs at time zero.

In freeze and thaw stability three QCs were prepared and kept at −20 °C. Twenty samples of each concentration were prepared. Five samples from each QCs were analysed on first day that were coded as the zero-hour time samples. The rest of the samples were frozen at −20°. After 24 hours (Cycle 1) five samples from each QCs were thawed and analysed, while the rest of the samples were frozen again at −20 °C. This procedure was repeated at 48 (Cycle 2) and 72 hours (Cycle 3). The integrity of drug was determined with respect to % recovery (Table 2).

**Table 2:** Auto-sampler, long term storage and freeze-thaw stability of Eperisone in plasma.

Conc. Level	QCL (n = 3)		QCM (n = 3)		QCH (n = 3)	
Time	Accuracy	Recovery (%)	Accuracy	% Recovery	Accuracy	Recovery %
***Auto sampler stability***						
*0* *hr*	101.03 ± 3.20	—	99.37 ± 3.81	—	95.2 ± 3.49	—
*6* *hr*	99.25 ± 1.46	98.23	97.77 ± 3.07	99.55	94.0 ± 4.01	106.29
*Mean Recovery (%)*				*101*.*36*		
***Long term stability***						
*Week 0*	103.80 ± 6.64	—	104.67 ± 5.13	—	99.33 ± 2.52	—
*Week 1*	101.67 ± 4.04	97.94	103.50 ± 0.50	98.89	98.00 ± 4.36	98.66
*Week 2*	103.20 ± 6.66	99.42	101.00 ± 5.20	96.5	100.67 ± 1.53	101.34
*Week 3*	102.67 ± 6.03	98.91	101.33 ± 3.51	96.82	98.17 ± 2.57	98.83
*Week 4*	101.37 ± 7.64	97.66	101.83 ± 7.78	97.29	95.73 ± 0.75	96.38
*Mean Recovery (%)*				*98*.*22*		
***Freeze thaw stability***						
*0* *hr*	98.52 ± 2.61		99.75 ± 4.46		98.47 ± 2.14	
*24* *hr*	98.22 ± 3.97	99.69	98.67 ± 4.21	98.92	99.61 ± 2.30	101.15
*48* *hr*	97.12 ± 3.34	98.58	98.62 ± 3.68	98.87	100.05 ± 3.13	101.60
*QCL 72* *hr*	95.84 ± 5.06	97.27	98.11 ± 2.08	98.36	97.61 ± 1.30	99.12
*Mean Recovery (%)*				*99*.*32*		

Linearity. Following FDA guidelines, linearity was determined by preparing eight different dilutions of Eperisone in plasma (10, 6, 4, 2, 1, 0.5, 0.05, 0.01 ng/ml) using a stock solution. These spiked dilutions were analysed and the responses were plotted against the respective theoretical concentrations. Analysis of the regression line was statistically performed and the coefficient of correlation, Y-intercept, slope of the regression line and residual sum of squares were estimated using *Microsoft Excel 2016* software. All concentrations were back calculated by weighted least squares regression (1/x) method and % accuracy of each concentration was also calculated.

Accuracy and Precision. In order to determine accuracy and precision, three QCs (QCL, QCM, QCH) were extracted from plasma. Five replicates of each concentration were injected each day and each concentration was back calculated with the help of the standard curve. Precision of QCs samples along with LLOQ were estimated from the standard curve for three days (Table 3).

**Table 3:** Intra and inter-day accuracy and precision of Eperisone quality control and calibration curve samples in plasma.

Quality Control samples	Conc. Level (ng/ml)	Intra-Day			Inter-Day		
		Mean ± S.D	Accuracy (%)	Precision (%)	Mean ± S.D	Accuracy (%)	Precision (%)
*LLOQ*	0.01	0.0096 ± 0.0002	96.15	8.31	0.0096 ± 0.0008	95.70	9.24
*QCL*	0.40	0.3990 ± 0.0156	99.75	3.31	0.3970 ± 0.0020	99.23	3.91
*QCM*	3.00	2.9514 ± 0.0217	98.22	3.27	2.9470 ± 0.0252	97.72	3.82
*QCH*	8.00	7.9614 ± 0.0978	99.52	1.35	7.9600 ± 0.1200	99.51	1.10
**Calibration Curve sample***s*	10	9.9333 ± 0.0868	99.33	0.66	9.8540 ± 0.0654	98.54	3.31
	6	6.0567 ± 0.0131	102.50	2.00	6.1500 ± 0.1231	100.94	3.27
	4	4.0460 ± 0.0131	101.15	1.10	4.0440 ± 0.1031	101.10	2.55
	2	2.0087 ± 0.0311	100.60	5.05	2.0120 ± 0.1016	100.43	9.24
	1	1.0230 ± 0.0156	103.30	3.31	1.0330 ± 0.0524	102.3	5.07
	0.5	0.4882 ± 0.0138	97.64	3.27	0.4788 ± 0.0365	95.76	7.61
	0.05	0.0479 ± 0.0008	95.73	1.10	0.0470 ± 0.0066	94.00	4.03
	0.01	0.0091 ± 0.0003	91.11	9.00	0.0091 ± 0.0004	90.06	9.66

Limit of quantification (LOQ) and limit of detection (LOD). LOQ and LOD were determined by analysing five samples of different concentrations i.e. 0.006, 0.01, 0.05, 0.5, 1, 2, 4, 6 & 10 ng/ml. The concentrations were estimated by back calculation from the calibration curve and the accuracy of each concentration was determined.

Analytical recovery and matrix effect. Analytical recovery (as given in Eq. 1) was estimated by comparing response of drug in mobile phase with the response of drug from extracted plasma considering the dilution factor.

1 $$Percent\,{Recovery}=\tfrac{Peak\,Area\,of\,Drug\,in\,Plasma}{Peak\,Area\,of\,Drug\,in\,Mobile\,Phase\times Dilution\,Factor}\times 100\,$$

Matrix effect was also evaluated by comparing the response of the QCs prepared in mobile phase and dried plasma matrix spiked with the same QCs concentration as that in mobile phase. The matrix factor is calculated by taking the ratio of response of QC in mobile phase to that of response of QC in spiked dried matrix. If the value of matrix factor is 1, it suggests no ion suppression or enhancement.

2 $$Matrix\,Factor=\tfrac{Peak\,area\,of\,dried\,matrix\,spiked\,with\,QCs\,concentration}{Peak\,Area\,of\,QCs\,in\,mobile\,phase}$$

### Pharmacokinetic study

A comparative PK study was conducted in a hospital set up to compare PK variables and parameters of oral controlled release osmotic pump (test) containing 150 mg Eperisone with three oral doses of the immediate release tablet (Smur containing 50 mg of Eperisone × 3 tablets), which was taken as reference.

#### Subjects, their inclusion and exclusion criteria

In this study twelve (12) healthy male human volunteers (coded as V1–V12) having age 18–24 years, weighing 67–72 Kg and possessing BMI 21.5–24.9 Kg/m^2^ were selected and enrolled (Table 4). The health status of all subjects was analysed by physical examination (blood pressure, weight, height, chest x-ray) and biochemical laboratory tests like CBC (complete blood cell), LFT (liver function test), and urine DR (diagnostic report). Subjects whose clinical laboratory results were not satisfactory according to the normal standard limits were excluded from the study. The subjects were also excluded if they fall under any of the following criteria: smoking more than 10 cigarettes per day; alcohol consumers or taking nicotine products; facing any type of acute infection within two weeks prior to study; having any chronic disease (which possibly may alter the pharmacokinetic results); enrolled in any other PK study within three months prior to the current study; donated blood within last thirty days before the current study; known allergic response to Eperisone; cannot avoid ingestion of grape fruit, cannot avoid beverages two days prior and during the study; having systolic blood pressure ≥150 mm Hg or ≤90 mm Hg; having diastolic blood pressure ≥100 mm Hg or ≤50 mmHg.

**Table 4:** Baseline demographic characteristics of 12 healthy human subjects and sequence of reference (A) and test (B) products administration.

S. No	Volunteer Code	Sequence	Age (Yrs)	Weight (Kg)	Height cm	BMI Kg/m^2^
1	V1	AB	23	66	175.3	21.5
2	V2	BA	21	67	167.6	23.8
3	V3	AB	20	70	172.7	23.5
4	V4	BA	24	67	170.2	23.1
5	V5	AB	24	68	165.1	24.9
6	V6	BA	22	64	170.2	22.1
7	V7	AB	22	69	175.3	22.5
8	V8	BA	22	71	177.8	22.5
9	V9	AB	18	66	170.2	22.8
10	V10	BA	24	72	175.3	23.4
11	V11	AB	21	67	172.7	22.5
12	V12	BA	23	63	167.6	21

#### Ethical Approval and Informed consent

The study protocol was approved by the Institutional Bioethics Committee (IBC), University of Karachi, Karachi, Pakistan with an approval no (IBCPH-07). The IBC had given the project approval as per the International Council for Harmonisation (ICH) Good Clinical Practice guidelines in accordance with the ethical principles provided in the current version of the Declaration of Helsinki^27^.

As per the ICH guidelines, all subjects were informed verbally and in writing about the consequences and possible outcomes of the study. A written consent form both in English and National (Urdu) languages were signed by each subject participating in the study and these forms were collected before the study. Volunteers were informed about the detail of the study, risks associated with participation and information regarding the right to withdraw at any time from participation without jeopardy.

#### Study design

The comparative PK study was designed as a single dose, two period, two treatment, two sequence, open label, randomized cross over. All volunteers were fasted overnight (at least 10 hours) before administration of the test (osmotic tablet) or the reference (immediate release tablet) product^28^. The immediate release dosage form (reference) was coded as “A” and osmotic dosage form (test) was coded as “B”. The study was conducted in two phases and each volunteer randomly received reference (A) and test (B) product with 240 ml of drinking water. A time period of two weeks was selected as a washout period between two treatments. The subjects were allowed to drink water and eat standardized (according to FDA specifications) meal 4 hours after administration of the drug. The sequence of administration of test (B) and reference (A) products is given in Table 4. A blood sample (5 ml) from each volunteer receiving immediate release product was drawn before administration (0 hours) and after dosing blood samples were drawn at time 0.5, 0.75, 1, 1.5, 2, 2.5,3, 4, 6, 8, 10 and 12 hours. Similarly, in case of volunteers receiving osmotic tablet a blood sample of 5 ml was withdrawn from each subject before the administration of osmotic tablet and after dosing blood samples were drawn at time 0.5,1,1.5,2,3,4,6,8,10,12,16,18,24 hours. Samples were collected into heparinized tubes. Plasma was separated at normal room temperature within 30 minutes of sample collection by centrifugation of the samples at 4000 rpm for 10 min. The separated plasma was transferred into polypropylene tubes and were stored at ≤−20 °C till analysed.

#### Tolerability assessment

During the study any drug related adverse effects in enrolled subjects were assessed by physical examination, vital signs observation and other clinical tests. Subjects were encouraged to report any unusual event immediately.

#### Pharmacokinetic variables and parameters

The data (concentration at different time points) obtained from bio-analytical study was used to calculate the PK parameters of osmotic and immediate release dosage forms of Eperisone. Non-compartmental analysis was applied by using a software Kinetica version 5.1 (Thermoelectron corp., Waltham, USA) to calculate various PK variables and parameters like maximum plasma concentration (C_max_) of drug, time to reach maximum plasma concentration (T_max_), area under plasma concentration-time curve (AUC), terminal half-life (t_1/2λz_), apparent volume of distribution during terminal phase after oral administration (V_z_/F), apparent clearance of drug after oral administration (Cl/F) and mean transit time (MTT). The relative bioavailability of test product (Osmotic pump) was also calculated by taking the percentage of the ratio between mean AUC_0-∞_ of test (osmotic tablet) and reference (immediate release) products.

#### Statistical analysis

As per FDA guidelines different parameters like subject effect nested, sequence effect and period effect were determined by Latin ANOVA (two-way) using a software Kinetica version 5.1. The PK parameter analysis was performed for logarithmic transformed data as per FDA guidelines^29^. Two-one-sided t test was used to compare PK variables and parameters of immediate and osmotic dosage forms using software Kinetica version 5.1. The 90% confidence limits were determined based on ratio of test and reference geometric mean. The PK variables and parameters of test and reference products will be equivalent, if logarithmic transformed data ratio of the lower t value is not less than 0.8 and upper t value is not greater than 1.25.

## Results and Discussion

### Method validation

**Figure 3:**
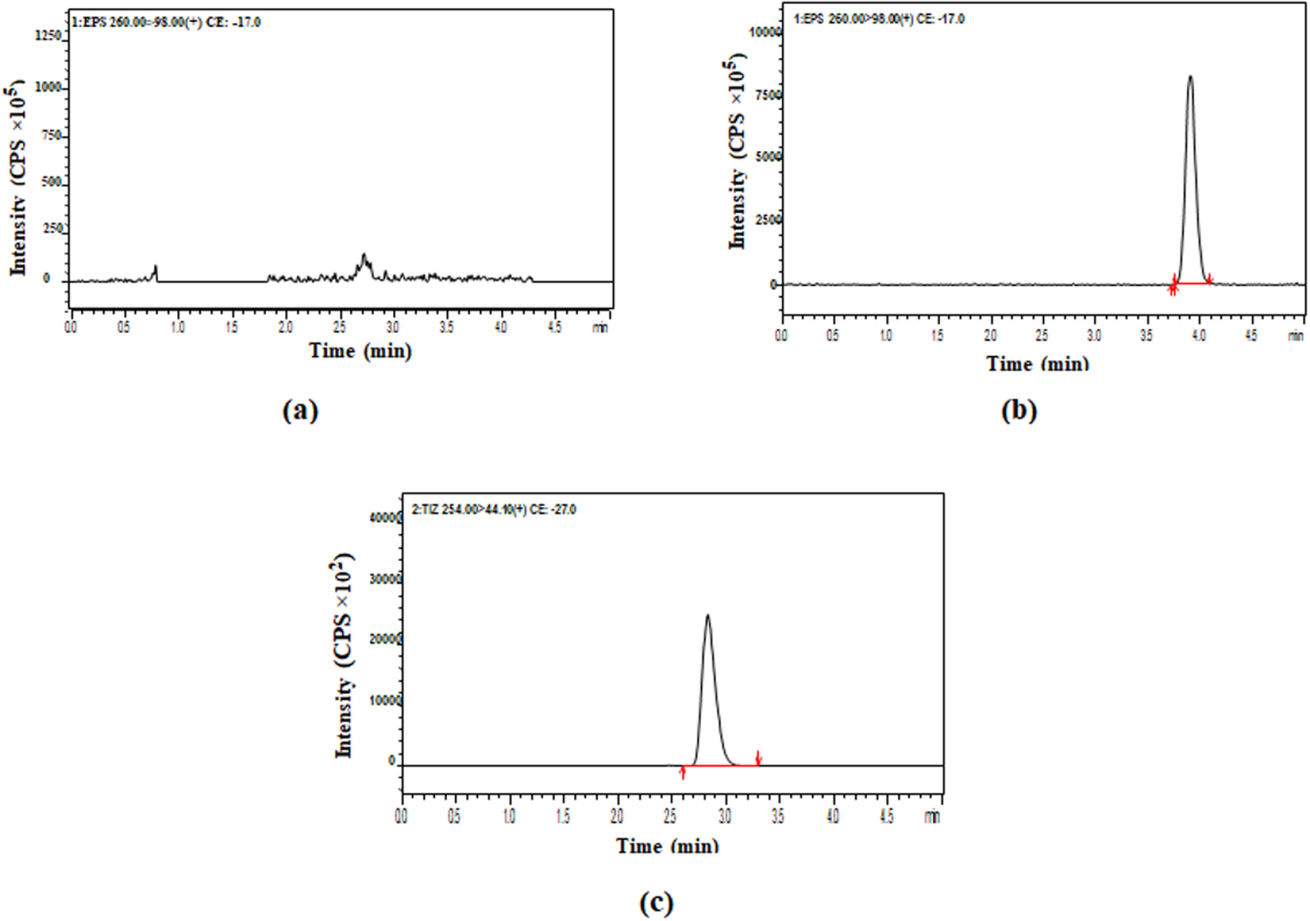
Chromatograms of (**a**) Blank Plasma (**b**) Eperisone at a concentration of 1 ng/ml in spiked plasma and (**c**) Tizanidine (IS).

The liquid chromatography-electrospray ionization-mass spectrometry (LC-ESI-MS/MS) method was efficiently applied for estimation of Eperisone in plasma using Tizanidine as internal standard (IS). Positive ionization was selected and mass transitions of Eperisone and Tizanidine were selected as 260.2 → 98.1 m/z and 254 → 44 m/z, sequentially (as shown in Figs. 1 and 2). Mobile phase containing 1% v/v acetic acid in deionized water and 1% v/v acetic acid in methanol in a ratio of 50:50 was pumped at a flow rate of 0.2 ml/min. The mean retention time of Eperisone and Tizanidine was 3.8 and 2.8 minutes (shown in Fig. 3), respectively. Simple protein precipitation technique using acetonitrile was employed for extraction of Eperisone from plasma.

**Figure 4:**
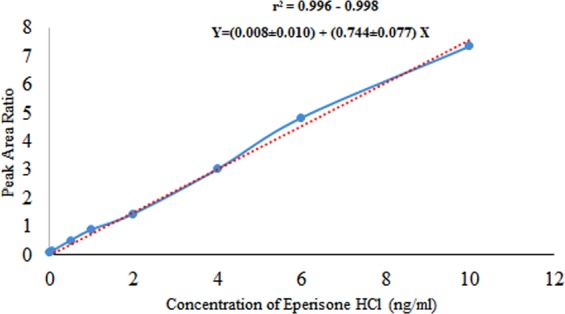
Calibration curve for the mean peak area ratio (from five runs of each concentration level) versus different Eperisone plasma concentration ranging from 0.01 to 10 ng/ml.

**Figure 5:**
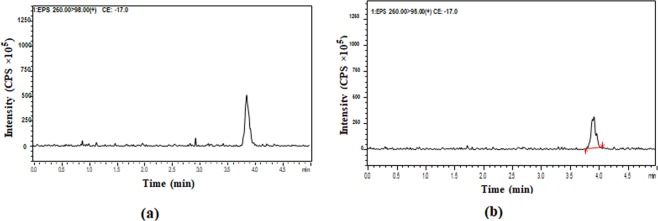
Chromatograms of Eperisone showing (**a**) Lower limit of quantification [LLOQ] and (**b**) Lower limit of detection [LOD].

The bio-analytical method validation was carried out by following FDA guidelines. The method was found selective for Eperisone estimation and practically matrix effect was found to be insignificant (matrix factor = 0.989–0.999). Linearity having a coefficient of correlation (r^2^) value 0.996–0.998 was obtained in a concentration range of 0.01 ng/ml to 10 ng/ml in plasma and the mean regression equation was calculated as Y = (0.008 ± 0.010) + (0.744 ± 0.077) X as shown in Fig. 4. Accuracy in plasma ranged from 90.03% to 111.16%. The mean analytical recovery from plasma was 91.6% (QCL), 91.94% (QCM) and 96.59% (QCH). The auto sampler stability of Eperisone was determined by spiking QCs, shown in Table 2. The mean recovery of Eperisone in auto sampler stability was 101.36%. The stock solution stability of Eperisone and internal standard was determined for four consecutive weeks, indicated in Table 1. The % recovery of Eperisone and IS was found to be 96.15–98.2% and 96.43–98.23%, respectively. The freeze thaw stability results are shown in Table 2 and the mean % recovery was 99.32%. In long term stability testing the mean % recovery of Eperisone in plasma was 98.22% as shown in Table 2. The results of intra and inter-day accuracy and precision (at four different concentration levels, n = 5) are summarized in Table 3. The intra and inter-day accuracy were found to be in a range of 93.6%–99.78% and 95.70–99.51%, respectively. The intra and inter- day precision were found to be in a range of 0.63–11.36% and 1.1–9.34%, respectively. The lower limit of quantification (LLOQ) and lower limit of detection (LLOD) were found to be 0.01 ng/ml and 0.006 ng/ml, respectively as shown in Fig. 5.

The results of bio-analytical method validation for estimation of Eperisone plasma concentration showed that the method was selective, linear, accurate and precise (as per FDA specifications). Several bio-analytical methods for estimation of Eperisone have been previously reported. The Meilli *et al*. reported a sensitive liquid chromatography–electrospray ionization-mass spectrometry (LC-ESI-MS) method for estimation of Eperisone in plasma with 0.01 ng/ml as a lower limit of quantification. In the study liquid extraction method was used and Tolperisone was selected as internal standard. The analysis of Eperisone was carried out at positive ion mode and the target and the product ions were set at 260 m/z and 98.2 m/z, respectively. Similar LC-ESI-MS method was reported by Jeoung *et al*. for determination of Eperisone in human plasma using a solvent extraction technique and Tolperisone as internal standard. The limit of detection for analyte was found to be 0.1 pg/ml for Eperisone with a linear range from 0.01 to 10.0 ng/ml [9]. Ding *et al*. also utilized the liquid extraction technique and similar LC-ESI-MS conditions for detection of Eperisone in human plasma. Miura *et al*. reported a method for rapid analysis of Eperisone, Tolperisone and Tizanidine in human plasma by LC-MS/MS technique. The precursor and product ions selected for Eperisone and Tizanidine were 260.2 → 98.1 m/z and 254.0 → 44.1 m/z, sequentially. The LOD was found to be 0.5 ng/ml with a linearity in the range of 1–500.0 ng/ml (r^2^ = 0.999), for Eperisone, Tolperisone and Tizanidine.

The previously reported methods were found to determine the concentration of Eperisone in plasma by utilizing complex liquid extraction technique with a lower limit of quantification of 0.01 ng/ml. The present method was found to be sensitive, simple and less time consuming because complex liquid extraction technique was not involved and Eperisone was extracted from plasma by simple protein precipitation using acetonitrile. The present method of Eperisone estimation was found to show a linearity with a coefficient of correlation (r^2^) value 0.996–0.998 in a concentration range of 0.01 ng/ml to 10 ng/ml. The mean retention time of Eperisone in present study was 3.8 minutes with 0.01 ng/ml and 0.006 ng/ml as LLOQ and LOD, respectively.

### Pharmacokinetic analysis

**Figure 6:**
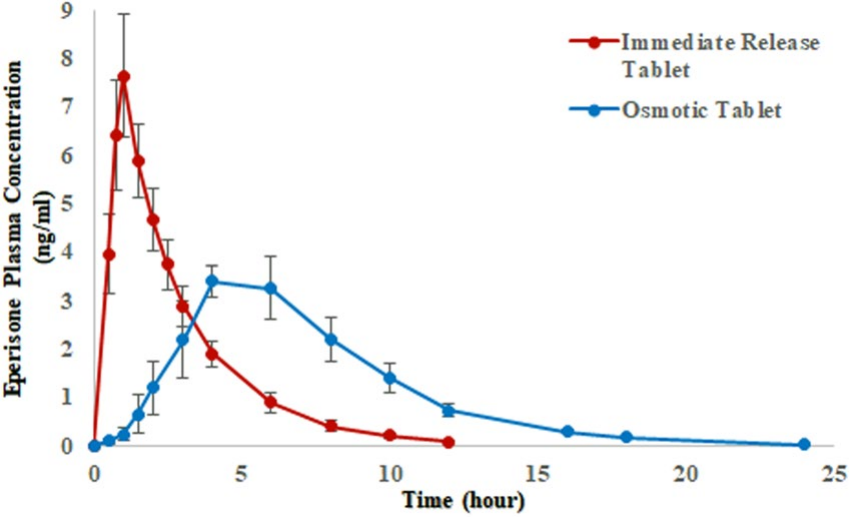
Mean plasma concentration versus time profiles obtained after administration of Eperisone 150 mg CR osmotic and immediate release tablets in 12 healthy subjects.

The mean plasma concentration versus time plot obtained after oral administration of 150 mg Eperisone as immediate release (A, reference) and osmotic dosage form (B, test) in 12 healthy human volunteers (fasted state) are shown in Fig. 6. Pharmacokinetic characteristics of Eperisone as summarized in Table 5 were obtained by non-compartmental analysis using PK software Kinetica version 5.1. The C_max_, T_max_, AUC_0-∞_, Cl/F and V_z_/F for immediate release tablets containing 150 mg Eperisone (as shown in Table 5) were 7.65 ± 1.28 ng/ml (5.80–9.95 ng/ml), 1.02 ± 0.24 h (0.75–1.50 h), 20.88 ± 1.33 ng/ml × h (18.96–23.58 ng/ml × h), 7211 ± 450 L/h (6361–7911 L/h) and 18800 ± 1690 L (16815–22755 L), respectively. Whereas, in case of osmotic tablets containing 150 mg Eperisone the C_max_, T_max_, AUC_0-∞_, Cl/F and V_z_/F were found to be 3.46 ± 0.33 ng/ml (2.95–4.01 ng/ml), 4.83 ± 1.21 h (3–6 h), 22.91 ± 1.67 ng/ml × h (19.39–25.22 ng/ml × h), 6585 ± 522 L/h (5948–7735 L/h) and 18982 ± 2491 L (15054–24100 L), sequentially. The AUC_0-t_, t _1/2λz_ and MTT of Eperisone obtained after ingestion of immediate release tablets as shown in Table 5 were found to be 22.67 ± 1.32 ng/ml × h (18.69–23.34 ng/ml × h), 1.81 ± 0.09 h (1.67–1.99) and 2.94 ± 0.26 h (2.56–3.39), respectively. The AUC_0-t,_ t_1/2λz_ and MTT of Eperisone obtained after ingestion of osmotic tablet as shown in Table 5 were found to be 22.85 ± 1.67 ng/ml × h (19.32–25.17 ng/ml × h), 1.99 ± 0.15 h (1.69–2.17) and 7.24 ± 0.42 h (6.51–7.78), respectively. The relative bioavailability of test product (Osmotic pump) calculated on the basis of AUC_0-∞_ of test and reference (immediate release) products was 109.7%. Numerious PK studies of Eperisone were reported by different investigators. A PK study, reported by Jeoung *et al*. in which 100 mg Eperisone was administered as immediate release tablets (each tablet contains 50 mg) in fasted state. The study reported the values of C_max_, T_max_ and AUC_0-∞_ as 1.25 ± 0.59 ng/ml, 1.28 ± 0.64 h and 4.21 ± 0.41 ng/ml × h, respectively^16^. Kim *et al*. reported that the C_max_, T_max_, AUC_0-∞_, V_z_/F and Cl/F of Eperisone were found to be 11.8 (0.8–44.8) ng/ml, 1.0 (0.5–2.0) h and 31.3 (2.5–76.1) ng/ml × h, 115,152 (23,744–281,751) L and 11,477 (1,855–56,785) L/h, respectively after administration of 150 mg Eperisone as a single dose^19^. A study conducted by Tanaka reported the C_max_, T_max_ and AUC_0-∞_ as 7.5–7.9 ng/ml, 1.6–1.9 h, 19.7–21.1 ng/ml × h, respectively after administration of 150 mg Eperisone as a single dose in fasted state^21^. Ryu *et al*. conducted a study in which Eperisone 75 mg as sustained release tablet was administered to each volunteer in fasted state and the values of C_max_, T_max_ and AUC_0-∞_ were reported as 0.743 (0.117–7.67) ng/ml, 1.0 (0.3–6.0) h and 3.731 (1.17–37.00) ng/ml × h, respectively^20^. Several other studies also reported the pharmacokinetic characteristics of Eperisone after per oral intake. After analysing the results of different Eperisone PK studies, it was noticed that the PK variables like C_max_, AUC_0-∞_ and PK parameters like volume of distribution and clearance values of Eperisone showed a significant variation between the study results. Interpersonal PK variation was also noticed (specifically in case of C_max_) within a study even after administration of Eperisone as sustained release product. The Eperisone was found to undergo extensive first pass effect, resulting in reduced bioavailability. The interpersonal PK variation was supposed to arise due to variation in metabolic capability of every individual^20,30^. In the present study the results of osmotic tablets containing Eperisone showed C_max_ in the range of 2.95–4.01 ng/ml. This indicates that the osmotic tablets were not significantly affected by the interpersonal variation in comparison to immediate release and sustained release tablets. The Eperisone release from the dosage form have an impact on its PK characteristics. In case of immediate release tablet whole amount of Eperisone will be liberated in a short span of time as compare to osmotic tablets. As Eperisone undergoes extensive first pass effect, the amount of Eperisone available for metabolism and the quantity of metabolic enzymes will affect the pharmacokinetic characteristics of the drug. As the quantity of enzymes (metabolizing Eperisone) is limited so the amount of Eperisone exposed to enzymes may affect the extent of first pass metabolism. Hence, interpersonal PK variation was observed either due to variation in the quantity of Eperisone metabolizing enzymes or rate of Eperisone release from the product^20^. In the present study AUC_0-∞_ of Eperisone for osmotic tablets and immediate release was in the range of 18.96–25.22 ng/ml × h. The relative bioavailability of test product was 109.7%. The T_max_ of immediate release product as shown in Table 5 is around 1 hour, indicating quick absorption of drug. Whereas, a delayed T_max_ (3–6 hours) in case of osmotic pump was observed due to the slow and controlled release of drug from the product. The t_1/2λz_ of immediate release tablets and osmotic tablets as shown in Table 5 were found to be 1.81 ± 0.09 h (1.67–1.99) and 1.99 ± 0.15 h, respectively. Jeoung *et al*. conducted a PK study in which 100 mg Eperisone was administered and the t_1/2λz_ was reported as 3.16 ± 0.41 h. Most of the PK studies involving Eperisone reported the t_1/2λz_ in the range of 1.6–2.0 hours. The MTT of Eperisone was greater in case of osmotic pump (7.24 h) due to prolong release from the product as compared to the (2.94 h) immediate release product.

**Table 5:** Comparative pharmacokinetic characteristics of Eperisone 150 mg after administration of immediate release conventional tablets (Reference) and osmotic pump dosage form (Test) in 12 healthy human subjects with geometric mean ratio and 90% CIs.

Type of dosage Form		C_max_ (ng/ml)	T_max_ (hr)	AUC_0-t_ (h.ng/ml)	AUC_0-∞_ (h.ng/ml)	t_1/2ʎz_ (h)	V_z_/F (L)	Cl/F (L/h)	MTT (h)
Immediate release (Reference = R)	Mean	7.65	1.02	20.67	20.88	1.81	18800	7211	2.94
	SD	1.28	0.24	1.32	1.33	0.09	1690	450	0.26
	%CV	16.78	23.36	6.4	6.38	5.14	8.99	6.24	8.91
	Min	5.8	0.75	18.69	18.96	1.67	16815	6361	2.56
	Max.	9.95	1.5	23.34	23.58	1.99	22755	7911	3.39
Osmotic pump (Test = T)	Mean	3.46	4.83	22.85	22.91	1.99	18982	6585	7.24
	SD	0.33	1.21	1.67	1.67	0.15	2491	522	0.42
	%CV	9.49	25.1	7.32	7.29	7.57	13.12	7.93	5.86
	Min	2.95	3	19.32	19.39	1.69	15054	5948	6.51
	Max.	4.01	6	25.17	25.22	2.17	24100	7735	7.78
Geometric Mean Ratio(T/R)		0.46	4.69	1.1	1.08	1.1	0.91	1	2.47
90% CI		0.41–0.51	3.74–5.89	1.05–1.16	1.03–1.14	1.04–1.16	0.85–0.98	0.90–1.12	2.36–2.59

The comparative statistical evaluation of PK variables and parameters was performed by using Latin square ANOVA (two-way) and two-one-sided t test on logarithmic transformed pharmacokinetic data of immediate release and osmotic pump. The statistical results indicated that the influence of subject, sequence and period on PK characteristics was insignificant. The geometric mean ratios (90% CIs) of C_max_, T_max_, AUC_0-∞_ and AUC_0-t_ as mentioned in Table 5 were found to be 0.46 (0.41–0.51), 4.69 (3.74–5.89), 1.08 (1.03–1.14) and 1.10 (1.05–1.16), respectively. However, for t_1/2λz_, Cl/F, V_z_**/**F and MTT the geometric mean ratios (90% CIs) were found to be 0.91 (0.86–0.96), 0.91 (0.85–0.98), 1.00 (0.90–1.12) and 2.47 (2.36–2.59), respectively. The AUC_0-∞_, AUC_0-t_, t_1/2λz_, Cl/F and V_z_/F of Eperisone obtained from test and reference products were considered equivalent, because of having their 90% CIs within the specified limit (0.8–1.25). Whereas, the C_max_, T_max_ and MTT were found to be non-equivalent, because the 90% CIs of these variables were beyond the specified limit (0.8–1.25). On the basis of the relative bioavailability (109.7%) the test product (Osmotic pump) was found bioequivalent to the reference product (immediate release).

Any serious adverse event was not noticed in volunteers during the study. Only drowsiness was reported by subjects (n = 2), who received immediate release tablet and the effect was diminished after 2 hours of administration.

## Conclusion

A sensitive, simple and less time consuming bio-analytical method utilizing chromatography-electrospray ionization-mass spectrometry (LC-ESI-MS) technique for determination of Eperisone in human plasma was developed and validated. A comparative PK study conducted between osmotic tablet (test) and marketed (reference) immediate release tablet (Smur) using a single dose of 150 mg Eperisone indicated no significant difference in AUC_0-∞,_ AUC_0-t,_ V_z_/F, Cl/F and t_1/2λz_ of Eperisone. But a significant difference in the values of C_max_, T_max_ and MTTs of test and reference products was found. A greater fluctuation in plasma concentration of Eperisone after the administration of immediate release tablet was found in comparison to osmotic tablet. The osmotic device was found to possess a relative bioavailability of 109.7% with extended release of drug and almost similar inter personal pharmacokinetics. Thus, the osmotic pump of Eperisone can be used to provide therapeutic effect of the drug for an extended period with almost similar inter personal pharmacokinetics as compare to immediate release tablets.
